# Plasma Biomarker Analysis in Pediatric ARDS: Generating Future Framework from a Pilot Randomized Control Trial of Methylprednisolone: *A Framework for Identifying Plasma Biomarkers Related to Clinical Outcomes in Pediatric ARDS*

**DOI:** 10.3389/fped.2016.00031

**Published:** 2016-03-31

**Authors:** Dai Kimura, Jordy Saravia, Cynthia R. Rovnaghi, Gianfranco Umberto Meduri, Andreas Schwingshackl, Stephania A. Cormier, Kanwaljeet J. Anand

**Affiliations:** ^1^Department of Pediatrics, University of Tennessee Health Science Center, Memphis, TN, USA; ^2^Department of Pediatrics, Le Bonheur Children’s Hospital, Memphis, TN, USA; ^3^Children’s Foundation Research Institute at Le Bonheur Children’s Hospital, Memphis, TN, USA; ^4^Pain Neurobiology Laboratory, Stanford University, Palo Alto, CA, USA; ^5^Department of Internal Medicine, Memphis Veterans Affairs Medical Center, Memphis, TN, USA; ^6^Division of Pulmonary, Critical Care, and Sleep Medicine, Department of Internal Medicine, University of Tennessee Health Science Center, Memphis, TN, USA; ^7^Department of Pediatrics, University of California Los Angeles, Los Angeles, CA, USA; ^8^Department of Microbiology, Immunology and Biochemistry, University of Tennessee Health Science Center, Memphis, TN, USA; ^9^Department of Physiology, University of Tennessee Health Science Center, Memphis, TN, USA; ^10^Department of Pediatrics, Stanford University School of Medicine, Palo Alto, CA, USA; ^11^Department of Anesthesiology, Perioperative and Pain Medicine, Stanford University School of Medicine, Palo Alto, CA, USA

**Keywords:** ARDS, biomarker, pediatric, methylprednisolone, MMP-8, Ang-2, ICAM-1, sRAGE

## Abstract

**Objective:**

Lung injury activates multiple pro-inflammatory pathways, including neutrophils, epithelial, and endothelial injury, and coagulation factors leading to acute respiratory distress syndrome (ARDS). Low-dose methylprednisolone therapy (MPT) improved oxygenation and ventilation in early pediatric ARDS without altering duration of mechanical ventilation or mortality. We evaluated the effects of MPT on biomarkers of endothelial [Ang-2 and soluble intercellular adhesion molecule-1 (sICAM-1)] or epithelial [soluble receptor for activated glycation end products (sRAGE)] injury, neutrophil activation [matrix metalloproteinase-8 (MMP-8)], and coagulation (plasminogen activator inhibitor-1).

**Design:**

Double-blind, placebo-controlled randomized trial.

**Setting:**

Tertiary-care pediatric intensive care unit (ICU).

**Patients:**

Mechanically ventilated children (0–18 years) with early ARDS.

**Interventions:**

Blood samples were collected on days 0 (before MPT), 7, and 14 during low-dose MPT (*n* = 17) vs. placebo (*n* = 18) therapy. The MPT group received a 2-mg/kg loading dose followed by 1 mg/kg/day continuous infusions from days 1 to 7, tapered off over 7 days; placebo group received equivalent amounts of 0.9% saline. We analyzed plasma samples using a multiplex assay for five biomarkers of ARDS. Multiple regression models were constructed to predict associations between changes in biomarkers and the clinical outcomes reported earlier, including P/F ratio on days 8 and 9, plateau pressure on days 1 and 2, PaCO_2_ on days 2 and 3, racemic epinephrine following extubation, and supplemental oxygen at ICU discharge.

**Results:**

No differences occurred in biomarker concentrations between the groups on day 0. On day 7, reduction in MMP-8 levels (*p* = 0.0016) occurred in the MPT group, whereas increases in sICAM-1 levels (*p* = 0.0005) occurred in the placebo group (no increases in sICAM-1 in the MPT group). sRAGE levels decreased in both MPT and placebo groups (*p* < 0.0001) from day 0 to day 7. On day 7, sRAGE levels were positively correlated with MPT group PaO_2_/FiO_2_ ratios on day 8 (*r* = 0.93, *p* = 0.024). O_2_ requirements at ICU transfer positively correlated with day 7 MMP-8 (*r* = 0.85, *p* = 0.016) and Ang-2 levels (*r* = 0.79, *p* = 0.036) in the placebo group and inversely correlated with day 7 sICAM-1 levels (*r* = −0.91, *p* = 0.005) in the MPT group.

**Conclusion:**

Biomarkers selected from endothelial, epithelial, or intravascular factors can be correlated with clinical endpoints in pediatric ARDS. For example, MPT could reduce neutrophil activation (⇓MMP-8), decrease endothelial injury (⇔sICAM-1), and allow epithelial recovery (⇓sRAGE). Large ARDS clinical trials should develop similar frameworks.

**Trial Registration:**

https://clinicaltrials.gov, NCT01274260.

## Background

The pathogenesis of acute respiratory distress syndrome (ARDS) may result from dysregulated inflammation in the endothelial and epithelial spaces from pulmonary or systemic circulations (1). ARDS is associated with alveolar and interstitial edema, infiltration of inflammatory cells (neutrophils and macrophages) into the alveolar space due to vascular permeability, abnormal coagulation, and endothelial and alveolar epithelial injury (1). Corticosteroids may suppress this dysregulated inflammation in ARDS and thereby improve clinical outcomes (2). In adult patients with ARDS, early low-dose corticosteroid treatment showed promising results (3). However, the American College of Critical Care Medicine recommends the use of corticosteroids only for adult patients with moderate to severe ARDS resulting from community-acquired pneumonia (grade 2B). A single-center retrospective study reported that utilization of corticosteroids in pediatric ARDS was associated with worsening clinical outcomes (4). However, this study did not record the indications for corticosteroid administration and neither considered the length of therapy nor the need for tapering steroids to prevent rebound inflammation (5).

We published the first pilot randomized trial of low-dose methylprednisolone therapy (MPT) for early pediatric ARDS examining the feasibility of this therapy (6). We modified the protocol for administering MPT in adult ARDS (3), which includes the slow tapering of MPT dosage over 1 week to avoid rebound lung inflammation and systemic inflammatory response syndrome (SIRS) leading to recurrent respiratory failure (7). MPT therapy did not alter ventilator-free days or mortality but appeared to alter oxygenation and ventilation (6). We also examined different regulatory patterns of pro- and anti-inflammatory plasma cytokines in the MPT and placebo groups to identify several cytokines as potential predictors for alterations in clinical parameters of disease severity (8). Larger randomized controlled studies are warranted for investigating corticosteroid use in early pediatric ARDS.

Corticosteroids are widely used in pediatric patients with ARDS (4), but their effects on biomarker levels in ARDS remain unknown. We selected five plasma biomarkers signifying the endothelial [Ang-2 and soluble intercellular adhesion molecule-1 (sICAM-1)], epithelial [soluble receptor for activated glycation end products (sRAGE)], coagulation [plasminogen activator inhibitor (PAI-1)], and neutrophil [matrix metalloproteinase-8 (MMP-8)] factors contributing to the pathogenesis of ARDS. The main purpose of this pilot study was to generate a potential framework for studying ARDS pathogenesis in future clinical trials.

## Materials and Methods

### Clinical Trial

A double-blind, placebo-controlled randomized trial (https://clinicaltrial.gov, NCT01274260) was conducted in pediatric intensive care unit (PICU) in Le Bonheur children’s hospital in Memphis, TN, USA (6). The Institutional Review Board of University of Tennessee Health Science Center approved this study. Children (0–18 years) fulfilling the Berlin definition of ARDS and receiving mechanical ventilation were included in the study after informed consent. Patients receiving glucocorticoids chronically, terminally ill or in hospice care, immunosuppressed, with extensive burns, adrenal insufficiency, vasculitis, diffuse alveolar hemorrhage, invasive fungal infection, chronic liver disease or gastrointestinal bleed within the past 1 month, or conditions with estimated 6-month mortality of >50% were excluded from the study. The patients were randomized into low-dose methylprednisolone (MPT, *n* = 17, and no death) or placebo (*n* = 18 and two deaths) group. There were no significant differences in baseline characteristics including age, weight, gender, race, pediatric risk of mortality (PRISM) III score, pediatric index of mortality-2 score, P/F ratio, PEEP, tidal volume (milliliter per kilogram), mean airway pressure, blood gas pH, PaCO_2_, and the number of lobes affected on chest X-ray. Only exceptions were higher plateau pressure of mechanical ventilation (*p* = 0.006) and lower pediatric logistic organ dysfunction scores (*p* = 0.04) in the MPT group. The causes of ARDS were mainly primary ARDS (>3/4 of the patients in both groups). The most common cause was pneumonia (12 in MPT and 7 in the placebo), followed by aspiration (5 in MPT and 7 in the placebo), bronchiolitis (4 in MPT and 3 in the placebo), and near drowning (2 in MPT and 2 in the placebo). The patients in the MPT group received a 2 mg/kg MPT loading dose within 72 h after diagnosis of ARDS, followed by a 1 mg/kg/day continuous infusion from days 1 to 7, then tapered off over 7 days. If patients were extubated before day 7, the dosage protocol was advanced to day 7 followed by tapering. Patients were ventilated according to ARDSnet recommendations modified for children using pressure regulated volume control mode of Servo-i ventilators (Maquet) set at tidal volume of 6–8 ml/kg (ideal body weight if the patient is obese). All the patients finished the study. Two patients died in the placebo group and no one in the MPT (*p* = 0.15). The duration of mechanical ventilation was 9.74 ± 6.62 days in the placebo group and 9.59 ± 5.21 days in the MPT (*p* = 0.94). There were no significant differences in ventilator-free days or length of stay in intensive care unit (ICU) or hospital between the groups. The MPT group had lower PaCO_2_ values on days 2 and 3, higher pH on day 2, higher PaO_2_/FiO_2_ ratios on days 8 and 9, fewer patients required racemic epinephrine inhalation treatment for post-extubation stridor, and fewer patients required supplemental oxygen at ICU transfer. MPT was not associated with any adverse effects, including secondary bacterial infection, hyperglycemia, and hypertension monitored regularly.

### Plasma Samples

Blood samples were collected in lavender-top, EDTA-containing tubes at day 0 (before steroid administration) and day 7 during the randomized control trial. The blood samples were transported to our hospital central laboratory, centrifuged at 1,000 × *g* for 10 min at 4°C, and stored at −80°C until used.

### Biomarker Analysis

We analyzed these plasma samples for MMP-8, Ang-2, PAI-1, sICAM-1, and sRAGE using a multiplex assay (Millipore, Billerica, MA, USA, cat#HSP4MAG-63K, #HAGP1MAG-12K, #HSP1MAG-63K, #HSCRMAG-32K), according to the manufacturer’s instructions. A standard curve was prepared over concentrations of 0.14–100 ng/ml for MMP-8, 13.7–10,000 pg/ml for Ang-2, 12–50,000 pg/ml for PAI-1 and sRAGE, and 60–250,000 pg/ml for sICAM-1. All samples were run in duplicates, and the average concentrations were used for statistical analysis. Only a few samples showed the biomarker values below or above the limit of the standard curve (two samples were above the limit on day 0 and one sample on day 7 below the limit for MMP-8. One sample on day 7 was below the limit for sRAGE).

### Statistical Analysis

We used univariate (descriptive statistics and frequency distributions), bivariate (Fisher’s test for variability, *t*-tests, and Pearson’s correlations), and multivariate analyses (multivariate linear regressions using the least squares method) to evaluate the effects of MPT in pediatric ARDS. We used StatPlus (AnalystSoft, Inc.) for generating descriptive statistics and frequency distributions for all biomarker levels at days 0 and 7 for the placebo and MPT groups. We used Prism 6 (GraphPad Software, Inc.) for comparing between the randomized groups at days 0 and 7, and comparing between days 0 and 7 within the MPT or placebo groups. Either the unpaired two-tailed *t*-test (parametric) or the Mann–Whitney *U* test (non-parametric) was used for group comparisons, after comparing their variances by the *F* test.

A correlation matrix for pairwise associations of Pearson correlation coefficients was also generated with simultaneously run *t*-tests for pathway analysis of the measured mediators. We used pairs of variables demonstrating strong correlation coefficients (*R* ≥ 0.7, *p* ≤ 0.01) to build multivariate regression models for predicting the clinical outcomes reported earlier (6), namely, PaO_2_/FiO_2_ (P/F) ratio on days 8 and 9, plateau pressure on days 1 and 2, PaCO_2_ on days 2 and 3, need for racemic epinephrine following extubation, or supplemental oxygen at ICU discharge.

We created bivariate correlation matrices and bar graphs using raw data without adjustment or imputation. Rarely, imputation of missing biomarker values was necessary to build the multivariate regression models explaining or contributing to glucocorticoid treatment-associated clinical outcomes. Biomarker values falling above or below the extreme values of the standard curve were assigned the highest or lowest values on the generated standard curve. Since these were exploratory, hypothesis-generating analyses, we did not make corrections for multiple comparisons between groups.

## Results

### Baseline and Time-Dependent Changes in Plasma Biomarker Levels

**Figure 1:**
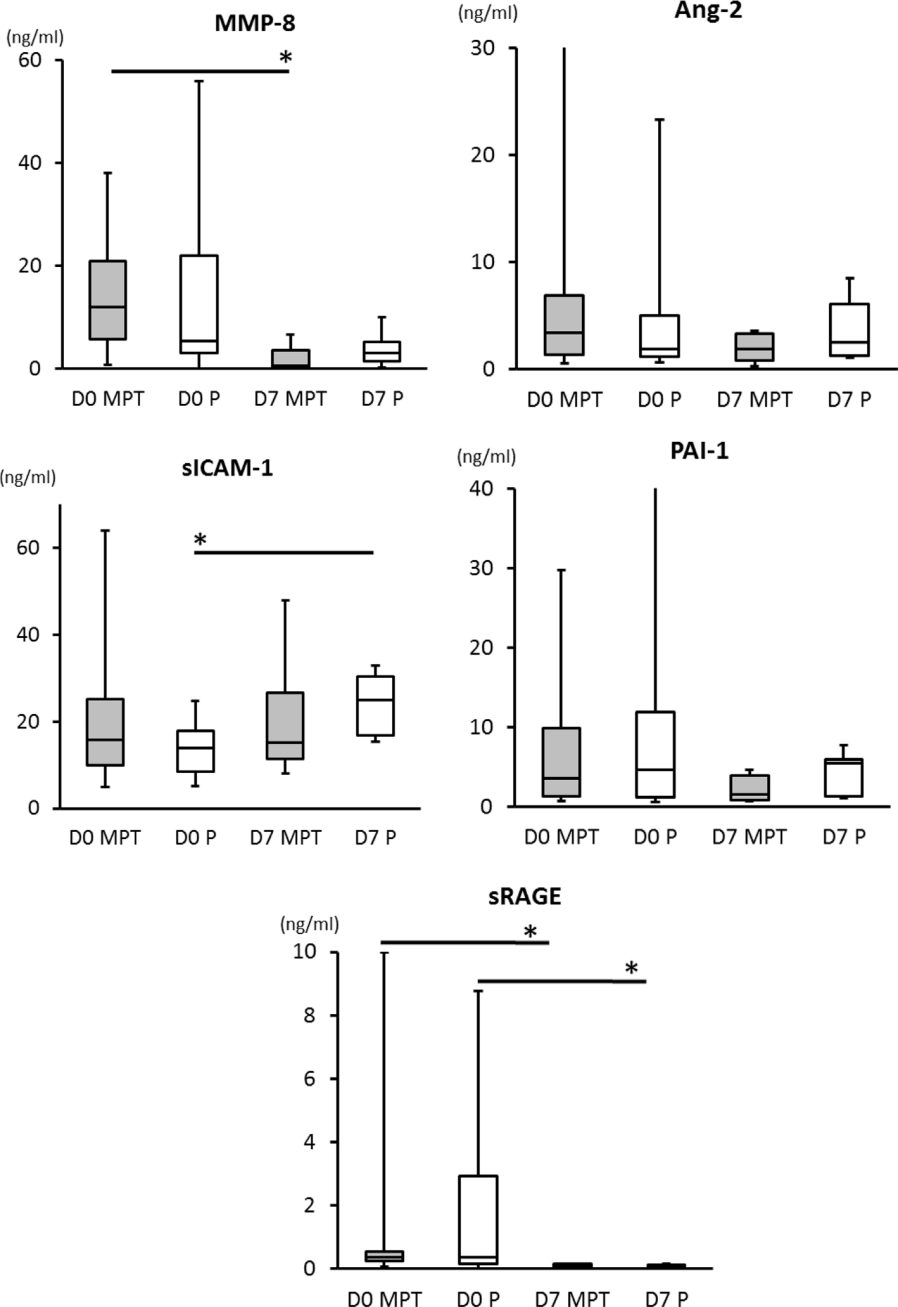
**Comparisons of biomarker levels between placebo and the MPT groups on days 0 and 7**. There are no differences between cytokine levels detected in MPT and control groups on day 0 or day 7. Two-tailed unpaired *t* or *u* test dependent were used dependent upon variances differences evaluated by *F* test. On day 7, we observed a significant reduction in plasma levels of MMP-8 (*p* = 0.0016) in the MPT and a significant increase in plasma levels of sICAM-1 (*p* = 0.0005) in the placebo group (MPT group had no increase by day 7). Levels of sRAGE decreased significantly in both the MPT and placebo groups (*p* < 0.002) from day 0 to day 7. A *p*-value of <0.05 was considered significant (*).

No differences occurred in the baseline concentrations of plasma biomarkers between the placebo and MPT groups on day 0 or on day 7. Within group, comparisons showed that MMP-8 levels decreased from day 0 to 7 (*p* = 0.0016) in the MPT group, but not in the placebo group. sICAM-1 levels increased significantly from day 0 to 7 (*p* = 0.0005) in the placebo group, but not in the MPT group. sRAGE levels decreased in both MPT and placebo groups (*p* < 0.0001) from day 0 to day 7 (Table 1). No significant differences occurred in Ang-2 and PAI-1 levels (Figure 1).

**Table 1:** **Significant results from comparison of biomarker levels within the placebo and the MPT groups between days 0 and 7**.

	*N*	Median	25% percentile	75% percentile	Mean	SD	SEM	Two-tailed Mann–Whitney	Two-tailed unpaired *t*-test
MPT, day 0: MMP-8 (ng/ml)	15	12	6.4	23	20	25	6.3	0.0016	
MPT, day 7: MMP-8 (ng/ml)	6	0.63	0.28	4.8	2.1	2.7	1.1		
MPT, day 0: sRAGE (pg/ml)	15	356	244	535	1,085	2,495	644	0.0007	
MPT, day 7: sRAGE (pg/ml)	6	74	16	136	79	62	25		
P, day 0: ICAM-1 (pg/ml)	18	13,814	8,444	17,783	13,973	5,453	1,285		0.0005
P, day 7: ICAM-1 (pg/ml)	7	24,824	16,710	30,346	24,461	6,859	2,592		
P, day 0: sRAGE (pg/ml)	18	376	152	2,941	1,599	2,346	553	0.0018	
P, day 7: sRAGE (pg/ml)	7	77	29	131	79	49	19		
A-S, day 0: MMP-8 (ng/ml)	32	10	3.9	23	19	25	4.4	0.0007	
A-S, day 7: MMP-8 (ng/ml)	13	1.9	0.47	5.1	3.1	3.1	0.86		
A-S, day 0: sRAGE (pg/ml)	33	363	196	1,209	1,365	2,390	416	<0.0001	
A-S, day 7: sRAGE (pg/ml)	13	77	26	127	79	53	15		

*Table of within-treatment group statistical differences, comparing days 0 to 7 levels of MMP-8, sRAGE, and ICAM-1. Day 0 compared to day 7 levels of PAI-1 and angiopoietin-2 did not differ. Methylprednisolone group (MPT), placebo group (P), all subjects (A-S), SD, and SEM*.

### Correlation between Biomarkers and Clinical Outcomes

Pearson’s correlation tests analyzed associations between biomarker levels and the clinical outcomes reported previously (6), including (1) P/F ratios on days 8 and 9, (2) mechanical ventilator plateau pressures on days 1 and 2, (3) PaCO_2_ values on days 2 and 3, (4) pH values on day 2, (5) need for racemic epinephrine following extubation, and (6) supplemental oxygen at PICU discharge (Table 2).

**Table 2:** **Pearson’s correlation table showing pairwise comparisons of clinical parameters of diseases and other factors**.

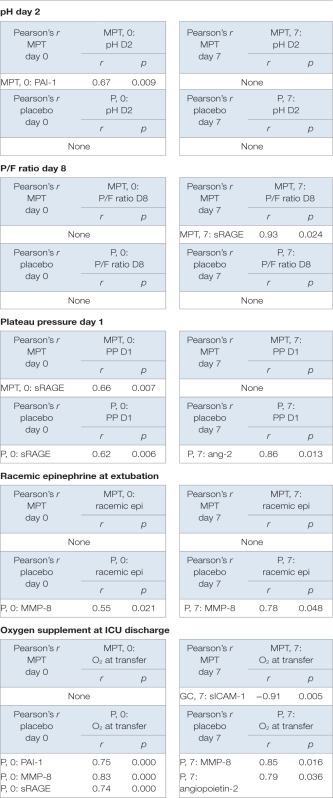

**r* is the Pearson’s correlation coefficient. A *p*-value of <0.05 was considered significant and was derived from a paired, two-tailed *t*-test. MPT group (day 0: biomarkers *n* = 14–15 and clinical parameters *n* = 17 and day 7: biomarkers *n* = 7 and clinical parameters *n* = 8). Placebo group (day 0: biomarkers *n* = 16–18 and clinical parameters *n* = 18 and day 7: biomarkers *n* = 7 and clinical parameters *n* = 7)*.

Soluble receptor for activated glycation end product levels (*r* = 0.93, *p* = 0.024) on day 7 correlated positively with improved oxygenation (PaO_2_/FiO_2_ ratios) on day 8 in the MPT group (Table 2), but not in the placebo group. Plateau pressures on day 1 were positively correlated with sRAGE on day 0 in both MPT (*r* = 0.66, *p* = 0.007), and placebo (*r* = 0.62, *p* = 0.006) groups. Plateau pressure on day 1 was also positively correlated with Ang-2 on day 7 in the placebo group (*r* = 0.86, *p* = 0.013), but not in the MPT group.

PaCO_2_ levels on days 2 and 3 did not correlate with any biomarker levels in either group. High pH on day 2 correlated with PAI-1 in the MPT group (*r* = 0.67, *p* = 0.009), but not in the placebo group. Racemic epinephrine therapy at extubation was positively correlated with MMP-8 in the placebo group on both day 0 and day 8 (*r* = 0.55, *p* = 0.021 and *r* = 0.76, *p* = 0.048, respectively), but not in the MPT group.

Supplemental oxygen at ICU transfer was inversely correlated with sICAM-1 on day 7 in the MPT group (*r* = −0.91, *p* = 0.005), but in the placebo group, it positively correlated with PAI-1 on day 0 (*r* = 0.75, *p* = 0.000), MMP-8 on day 7 (*r* = 0.85, *p* = 0.016), and Ang-2 on day 7 (*r* = 0.79, *p* = 0.036).

## Discussion

A randomized controlled trial of low-dose MPT for children with early pediatric ARDS (6) did not change mortality or morbidity (ventilator-free days at day 28) but altered the clinical parameters for oxygenation and ventilation. An observational study showed that 60% of pediatric patients received corticosteroids during the course of ARDS, and that exposure of corticosteroids for >24 h was independently associated with longer duration of mechanical ventilation (4). Those patients received corticosteroid with different regimens secondary to septic shock or other clinical indications. Thus, corticosteroid therapy for pediatric ARDS will remain controversial until larger controlled studies are performed. We propose that specific markers for endothelial or epithelial injury, coagulation and neutrophil activation, with known roles in the pathophysiology of ARDS, be selected as explanatory variables in future clinical trials.

In our study, MMP-8 levels decreased in the MPT group, but did not change in the placebo group. Also MMP-8 levels at days 0 and 7 positively correlated with supplemental O_2_ at transfer from PICU in the placebo group. The role of MMP-8 is not only extracellular matrix turnover but also positive modulation of inflammation and other physiological activities (9). In mice hyperoxia-induced acute lung injury model, MMP-8 was upregulated and seems to be produced in neutrophils, mesenchymal cell, and macrophages (10). In ventilator-induced lung injury model, absence of MMP-8 by knockout mice or specific inhibition of MMP-8 were associated with improved gas exchange, decreased lung edema and permeability, and decreased histological lung injury (11). Early elevations of MMP-8 in the bronchoalveolar lavage (BAL) fluid were associated with prolonged mechanical ventilation in pediatric ARDS (12), greater acute lung injury with higher PaO_2_/FiO_2_ ratios in adult patients (13), but did not predict their 90-day mortality (14). Overall decreases in MMP-8 levels seem to be associated with the reduction in local and systemic inflammation. In this study, MMP-8 levels did not decrease and were positively correlated with IL-6 and IL-10 on days 0 and 7 in the placebo group, possibly due to sustained active inflammation even on day 7 in the placebo group. Therefore, we can generate a hypothesis that MPT decreased lung inflammation *via* downregulation of MMP-8 in inflammatory cells, thus promoted early recovery from ARDS, and improved oxygenation in the recovery phase. This hypothesis needs to be validated in a future trial.

During sepsis or ARDS, Ang-2 is upregulated in the endothelium and believed to antagonize Ang-1. Upregulated Ang-2 is associated with impaired endothelial barrier function and increased adhesion and migration of inflammatory cells (15). Clinical studies in adults demonstrated higher plasma Ang-2 concentrations in ARDS patients (16), also associated with later development of ARDS (17), and ARDS mortality in adult patients (18, 19). Children with severe sepsis or septic shock have higher Ang-2 levels or Ang-2/Ang-1 ratios as compared to patients with SIRS (20). In this study, Ang-2 levels on day 7 correlated with plateau pressures on day 1 and the need for supplemental O_2_ at ICU discharge in the placebo group. This may indicate that acute lung injury on day 1 correlated with endothelial injury on day 7 and endothelial injury on day 7 correlated with oxygenation at ICU discharge. In the MPT group, Ang-2 levels on day 7 negatively correlated with IL-1RA. This may indicate that MPT may decrease endothelial injury *via* induction of anti-inflammatory cytokine, IL-1RA.

Soluble intercellular adhesion molecule-1 is an adhesion molecule expressed in the membrane of leukocytes, vascular endothelium, and alveolar epithelium (21). It is involved in neutrophil recruitment into the lung. In adults, plasma sICAM-1 levels appear to correlate with ARDS development (22, 23) and ARDS mortality (22, 24, 25). In pediatric ARDS, early elevation of sICAM-1 was associated with prolonged duration of mechanical ventilation and increased mortality (26, 27). In this study, sICAM-1 levels increased from day 0 to 7 in the placebo group, whereas in the MPT group sICAM-1 levels on day 7 were positive predictors for O_2_ supplement at transfer. This indicates that MPT may prevent endothelial injury, decrease permeability, and improve pulmonary edema leading to improved oxygenation. sICAM-1 levels correlated with Ang-2 levels on days 0 and 7, indicating similar roles as endothelial injury biomarkers. Interestingly, both sICAM-1 and Ang-2 levels in the placebo group had positive correlation with many cytokines on day 7. However, in the MPT group, Ang-2 and sICAM-1 on day 7 did not correlate with these cytokines. These findings may indicate that the endothelial injury underlying pediatric ARDS may continue until later phases of ARDS, dependent upon the degree of the inflammation, if not treated with corticosteroid.

Soluble receptor for activated glycation end products, the secreted forms of the epithelial RAGE membrane receptors, are multiligand-binding receptors that can bind advanced glycation end products, amyloid beta-peptide, S100 proteins, and high-mobility group box-1. It results in activation of the pro-inflammatory transcription factor nuclear factor-kB (NF-kB) (28). sRAGE levels in plasma and BAL were elevated in rat models of lung injury (29). In adult ARDS studies, its results are controversial. In adult ARDS, sRAGE level was associated with ARDS development and mortality (30, 31). However, in other studies, sRAGE did not show any association with the development of ARDS (17, 32, 33). Recent study demonstrated that serum sRAGE was elevated in children with severe bronchiolitis (34); however, there are no published data in pediatric ARDS. In this study, sRAGE levels decreased in all subjects, MPT, and the placebo groups from days 0 to 7. Furthermore, sRAGE levels on day 0 are predictors of higher plateau pressure on day 1 in both the groups. This indicates that higher plateau pressure correlated with more epithelial injury in early ARDS. However, by day 7, sRAGE levels decreased in both the MPT and placebo groups. Does this indicate that epithelial injury is more likely in the early stages of ARDS compared to the later stage? sRAGE levels on day 0 positively correlated with IL-6 levels in both the MPT and placebo groups, and this could be a manifestation of the pro-inflammatory effect of sRAGE.

Plasminogen activator inhibitor-1 is an anti-fibrinolytic agent. During ARDS, deposition of fibrin in the alveolar space is observed with disruption of the lung endothelial and alveolar epithelial barrier (35). Elevation of PAI-1 activity in plasma and air spaces associated with a decrease in alveolar fibrinolysis is observed in early ARDS (36). In a secondary analysis from adult ARDS patients, an elevated plasma PAI-1 level was significantly associated with poor oxygenation (37). Elevated plasma PAI-1 was also associated with mortality in pediatric ARDS (38). In this study, PAI-1 levels on day 0 correlated positively with high pH in the MPT group and were predictors of high pH on day 2 in our linear regression model. On day 0, PAI-1 level correlated positively with many cytokines and chemokines. This indicates that increased severity of inflammation may be associated with the deposition of fibrin in earlier stages of ARDS.

In adult ARDS, the same MPT protocol was associated with decreases in plasma IL-6 and proadrenomedullin levels and increases in protein C levels, compared to the placebo (39). As previously reported, MPT decreased IL-6 in pediatric ARDS as well (8). Considering the biomarker changes in this study, we propose that corticosteroids may attenuate inflammation, promote early recovery from the epithelial injury, and prevent sustained endothelial damage, leading to earlier improvement in pulmonary edema and oxygenation seen as clinical outcomes. This hypothesis should be tested in larger clinical trials of MPT in pediatric ARDS in the future.

This is the first study to measure plasma biomarkers for endothelial and epithelial injury at the different time points of pediatric ARDS. However, proteomic analysis methods have further advanced, and now it is technically possible to measure 1,310 protein levels simultaneously and precisely from just 150 μl of plasma samples (40, 41). Combinations of these biomarkers could discover novel therapeutic options and predictors of clinical outcomes in the future.

Limitations of this study include a relatively small number of samples; therefore, our results need to be validated in larger clinical trials. Our clinical trial was designed to demonstrate the feasibility of the MPT protocol in pediatric early ARDS. Our biomarker analysis was not the main purpose of the study and we could not conduct functional analyses. Also, we only obtained clinical samples on days 0 and 7, whereas the timing for peak levels of these biomarkers may be different. Finally, we did not conduct BAL fluid collection or lung biopsy, so our results may not represent local pulmonary the changes but may represent systemic changes.

## Conclusion

In this study, we demonstrated changes in selected biomarkers of ARDS between the placebo and MPT groups that were correlated with clinical endpoints. We cannot provide meaningful conclusions due to small sample size. However, we can generate a hypothesis that the anti-inflammatory effects of MPT may allow faster epithelial recovery manifested by decreases in sRAGE levels, thus avoiding sustained endothelial injury associated with elevated sICAM-1 levels in the placebo group. Future controlled studies with larger sample sizes should validate this hypothesis.

## Author Contributions

DK, SC, and KA: conception and design of study. DK: acquisition of data. DK, JS, CR, GM, AS, SC, and KA: analysis and interpretation of data and drafting and critically revising the manuscript. All the authors approved the final version of the manuscript.

## Conflict of Interest Statement

The authors declare that the research was conducted in the absence of any commercial or financial relationships that could be construed as a potential conflict of interest.

## Abbreviations

Ang-2, angiopoietin-2; ARDS, acute respiratory distress syndrome; ICU, intensive care unit; MMP-8, matrix metalloproteinase-8; MPT, methylprednisolone; PAI-1, plasminogen activator inhibitor-1; PICU, pediatric intensive care unit; sICAM-1, soluble intercellular adhesion molecule-1; SIRS, systemic inflammatory response syndrome; sRAGE, soluble receptor for activated glycation end products.
